# Supplementary material for: Improved drug-screening tests of candidate anti-cancer drugs in patient-derived xenografts through use of numerous measures of tumor growth determined in multiple independent laboratories
Source: PLoS One. 2025 Jun 18;20(6):e0324141. doi: 10.1371/journal.pone.0324141 (PMC12176186; doi:10.1371/journal.pone.0324141)
Supplement: S1 File — A step-by-step illustration of how the four screening tests are scored for an example data set. (DOCX) [file pone.0324141.s001.docx]

**S1 File. Example PDX statistical calculations**

The purpose of this supplement is to provide a step-by-step illustration of how the four screening tests are scored for an example data set.

We begin by providing notation for the Patient Derived Xenograft data that we use. The first table below, Table S1, consists of fictitious data, and is used as a means for illustrating the various values in the Notation section directly below.

*Notation*

For a given tumor model and a drug treatment, at each of *L lab*s, equal numbers (when possible) of PDX mice are generated and placed into two groups, although for our purposes, this condition is not necessary. The first group (treated) is administered the drug treatment on a fixed schedule (e.g., every three-four days). The second group (control) is fed no drug treatment.

Consider the following notation, applied to the standardized (i.e., tumor growth data transformed into percent-relative change in tumor volume relative to tumor volume at day 0 = %∆Vol(t)) empirical tumor growth data:

$g$ = The treatment group [$g=0$ (control), $g=1$ (treated)] to which a mouse has been assigned.

$l$ = The specific lab where the data have been generated. Note that $1\leq l\leq L$, where *L* is the total number of labs.

$i$ = The $i^{th}$mouse in a particular lab and treatment group. Note that $1\leq i\leq I\left( g,l \right)$, where $I\left( g,l \right)$ is the number of mice in a given treatment group *g* and lab *l*. When the values *g* and *l* are provided, this notation is reduced to *I*.

$t(g,l,i)$ = The day on which a specific tumor volume measurement is recorded for the $i^{th}$mouse in treatment group $g$ for lab *l*. When the values *g*, *i*, and *l* are provided, this notation is reduced to *t*.

Vol(t) is the tumor volume for a given mouse in a given group and lab.

%∆Vol(t), as mentioned above and defined in the main part of this work, is given by:

%∆Vol(t) = 100 × (Vol(t)- Vol(0))/Vol(0).

**Table S1**. Fictitious data to illustrate various notation terms.

| **Lab** | **Group** | **MouseID** | **Day (t)** | **Vol(t)** | **%∆Vol(t)** |
| --- | --- | --- | --- | --- | --- |
| Lab01 | 0 | Mouse01 | 0 | 327.00 | 0.00 |
|  |  |  | 4 | 692.40 | 111.74 |
|  |  |  | 9 | 1130.88 | 245.83 |
|  |  |  | 14 | 1657.06 | 406.74 |
|  |  |  | 17 | 2288.47 | 599.84 |
|  |  |  | NA | NA | NA |
|  |  | Mouse02 | 0 | 528.00 | 0.00 |
|  |  |  | 4 | 933.60 | 76.82 |
|  |  |  | 9 | 1420.32 | 169.00 |
|  |  |  | 14 | 2004.38 | 279.62 |
|  |  |  | 17 | 2705.26 | 412.36 |
|  |  |  | 21 | 3546.31 | 571.65 |
|  | 1 | Mouse01 | 0 | 1202.00 | 0.00 |
|  |  |  | 4 | 1091.60 | -9.18 |
|  |  |  | 9 | 1003.28 | -16.53 |
|  |  |  | 14 | 932.62 | -22.41 |
|  |  |  | 17 | 876.10 | -27.11 |
|  |  |  | 21 | 830.88 | -30.88 |
|  |  | Mouse02 | 0 | 300.00 | 0.00 |
|  |  |  | 6 | 195.00 | -35.00 |
|  |  |  | 10 | 126.75 | -57.75 |
|  |  |  | 15 | 82.39 | -72.54 |
|  |  |  | 18 | 53.55 | -82.15 |
|  |  |  | 22 | 34.81 | -88.40 |
| Lab02 | 0 | Mouse01 | 0 | 155.00 | 0.00 |
|  |  |  | 4 | 486.00 | 213.55 |
|  |  |  | 10 | 883.20 | 469.81 |
|  |  |  | 15 | 1359.84 | 777.32 |
|  |  |  | NA | NA | NA |
|  |  |  | NA | NA | NA |
|  |  | Mouse02 | 0 | 765.20 | 0.00 |
|  |  |  | 4 | 1218.24 | 59.21 |
|  |  |  | 9 | 1761.89 | 130.25 |
|  |  |  | 14 | 2414.27 | 215.51 |
|  |  |  | 17 | 3197.12 | 317.81 |
|  |  |  | 21 | 4136.54 | 440.58 |
|  | 1 | Mouse01 | 0 | 1301.00 | 0.00 |
|  |  |  | 4 | 1300.90 | -0.01 |
|  |  |  | 9 | 1300.81 | -0.01 |
|  |  |  | 14 | 1300.73 | -0.02 |
|  |  |  | 17 | 1300.66 | -0.03 |
|  |  |  | 21 | 1300.59 | -0.03 |
|  |  | Mouse02 | 0 | 427.60 | 0.00 |
|  |  |  | 6 | 226.63 | -47.00 |
|  |  |  | 10 | 120.11 | -71.91 |
|  |  |  | 15 | 63.66 | -85.11 |
|  |  |  | 18 | 33.74 | -92.11 |
|  |  |  | 22 | 17.88 | -95.82 |
| Lab03 | 0 | Mouse01 | 0 | 2322.00 | 0.00 |
|  |  |  | 4 | 3086.40 | 32.92 |
|  |  |  | 10 | 4003.68 | 72.42 |
|  |  |  | 15 | 5104.42 | 119.83 |
|  |  |  | 17 | 6425.30 | 176.71 |
|  |  |  | 21 | 8010.36 | 244.98 |
|  |  | Mouse02 | 0 | 677.00 | 0.00 |
|  |  |  | 4 | 1112.40 | 64.31 |
|  |  |  | 9 | 1634.88 | 141.49 |
|  |  |  | 14 | 2261.86 | 234.10 |
|  |  |  | 17 | 3014.23 | 345.23 |
|  |  |  | 21 | 3917.07 | 478.59 |
|  | 1 | Mouse01 | 0 | 111.00 | 0.00 |
|  |  |  | 4 | 88.80 | -20.00 |
|  |  |  | 9 | 71.04 | -36.00 |
|  |  |  | 14 | 56.83 | -48.80 |
|  |  |  | 17 | 45.47 | -59.04 |
|  |  |  | 21 | 36.37 | -67.23 |
|  |  | Mouse02 | 0 | 1000.00 | 0.00 |
|  |  |  | 6 | 710.00 | -29.00 |
|  |  |  | 10 | 504.10 | -49.59 |
|  |  |  | 13 | 357.91 | -64.21 |
|  |  |  | 18 | 254.12 | -74.59 |
|  |  |  | 22 | 180.42 | -81.96 |
|  |  | Mouse03 | 0 | 1000.00 | 0.00 |
|  |  |  | 7 | 710.00 | -29.00 |
|  |  |  | 10 | 504.10 | -49.59 |
|  |  |  | 15 | 357.91 | -64.21 |
|  |  |  | 19 | 254.12 | -74.59 |
|  |  |  | 21 | 180.42 | -81.96 |

The purpose of this table is to demonstrate that different mice can have tumor values recorded at different time points. Also, we can have different numbers of mice in the control and treated groups (See Lab 03 data).

In what follows, we demonstrate how to perform the screening test values using the Completely Responsive (CR) tumor type with data drawn from two labs (*L* = 2). The data are from the Evrard et al. publication. The PDX Trial Centers are Huntsman Cancer Institute/Baylor College of Medicine (HCI-BCM) and MD Anderson Cancer Center (MDACC).

**Table S2**. %ΔVol(t) values for control- and treated-mice that are randomly selected from the HCI-BCM and MDACC labs.

| **Lab** | **Group** | **Mouse** | **Day (t)** | **%ΔVol(t)** |
| --- | --- | --- | --- | --- |
| HCI-BCM | 0 | Mouse01 | 0 | 0 |
|  |  |  | 4 | -1.472 |
|  |  |  | 7 | 30.52 |
|  |  |  | 11 | 65.653 |
|  |  |  | 14 | 136.408 |
|  |  |  | 18 | 157.9 |
|  |  |  | 21 | 432.581 |
|  |  | Mouse02 | 0 | 0 |
|  |  |  | 4 | 4.427 |
|  |  |  | 7 | 55.846 |
|  |  |  | 11 | 69.807 |
|  |  |  | 14 | 124.801 |
|  |  |  | 18 | 230.988 |
|  |  |  | 21 | 370.431 |
|  |  | Mouse03 | 0 | 0 |
|  |  |  | 4 | 13.535 |
|  |  |  | 7 | 66.883 |
|  |  |  | 11 | 134.773 |
|  |  |  | 14 | 183.369 |
|  |  |  | 18 | 367.243 |
|  |  |  | 21 | 405.616 |
|  | 1 | Mouse01 | 0 | 0 |
|  |  |  | 4 | 9.375 |
|  |  |  | 7 | -50 |
|  |  |  | 11 | -78.038 |
|  |  |  | 14 | -100 |
|  |  |  | 18 | -100 |
|  |  |  | 21 | -100 |
|  |  | Mouse02 | 0 | 0 |
|  |  |  | 4 | 6.904 |
|  |  |  | 7 | -53.722 |
|  |  |  | 11 | -74.11 |
|  |  |  | 14 | -100 |
|  |  |  | 18 | -100 |
|  |  |  | 21 | -100 |
|  |  | Mouse03 | 0 | 0 |
|  |  |  | 4 | 59.159 |
|  |  |  | 7 | -44.244 |
|  |  |  | 11 | -75.676 |
|  |  |  | 14 | -87.988 |
|  |  |  | 18 | -100 |
|  |  |  | 21 | -100 |
|  | | | | |
| MDACC | 0 | Mouse01 | 0 | 0 |
|  |  |  | 4 | 101.091 |
|  |  |  | 7 | 176.176 |
|  |  |  | 11 | 334.944 |
|  |  |  | 14 | 445.536 |
|  |  |  | 18 | 460.349 |
|  |  |  | 21 | 517.302 |
|  |  | Mouse02 | 0 | 0 |
|  |  |  | 4 | 22.356 |
|  |  |  | 7 | 79.041 |
|  |  |  | 11 | 153.702 |
|  |  |  | 14 | 232.733 |
|  |  |  | 18 | 370.698 |
|  |  |  | 21 | 425.392 |
|  |  | Mouse03 | 0 | 0 |
|  |  |  | 4 | 42.647 |
|  |  |  | 7 | 47.045 |
|  |  |  | 11 | 136.869 |
|  |  |  | 14 | 191.363 |
|  |  |  | 18 | 312.123 |
|  |  |  | 21 | 356.102 |
|  | 1 | Mouse01 | 0 | 0 |
|  |  |  | 4 | 45.11 |
|  |  |  | 7 | -20.857 |
|  |  |  | 11 | -68.084 |
|  |  |  | 14 | -79.39 |
|  |  |  | 18 | -93.044 |
|  |  |  | 21 | -100 |
|  |  | Mouse02 | 0 | 0 |
|  |  |  | 4 | 32.251 |
|  |  |  | 7 | -51.422 |
|  |  |  | 11 | -77.187 |
|  |  |  | 14 | -92.301 |
|  |  |  | 18 | -97.719 |
|  |  |  | 21 | -100 |
|  |  | Mouse03 | 0 | 0 |
|  |  |  | 4 | 15.827 |
|  |  |  | 7 | -65.326 |
|  |  |  | 11 | -80.572 |
|  |  |  | 14 | -97.572 |
|  |  |  | 18 | -97.572 |
|  |  |  | 21 | -100 |

To be consistent with the notation in the main paper, we refer to Trial Centers as labs from this point forward. Scatter-plot representation of these data are presented in Figures S1 and S2 (HCI-BCM and MDACC).

For our CR tumor model with *L* = 2, *l* = 1 is HCI-BCM and *l* = 2 is MDACC.

Also, $I\left( g,l \right)$ = 3 for both groups and for both labs.

*Comprehensive computation of four screening tests*

Here we provide a step-by-step description for determining the four screening-test outcomes Single-Measure-Single-Lab (SMSL), Numerous-Measures-Single-Lab (NMSL), Single-Measure-Numerous-Labs (SMNL), and Numerous-Measures-Numerous-Labs (NMNL). These screening tests are defined in the main body of the paper. The step-by-step process may be repeated for any data set where we have a basis table (defined in the main body of the paper).

**Figure S1**. A scatter plot of the Table S2 values for the HCI-BCM lab (*l* = 1). Each line represents the Empirical Tumor Growth Trajectory for a given mouse. Trajectories for the control mice (*g* = 0) are represented by solid lines, and for the treated mice (*g* = 1) by dashed lines.


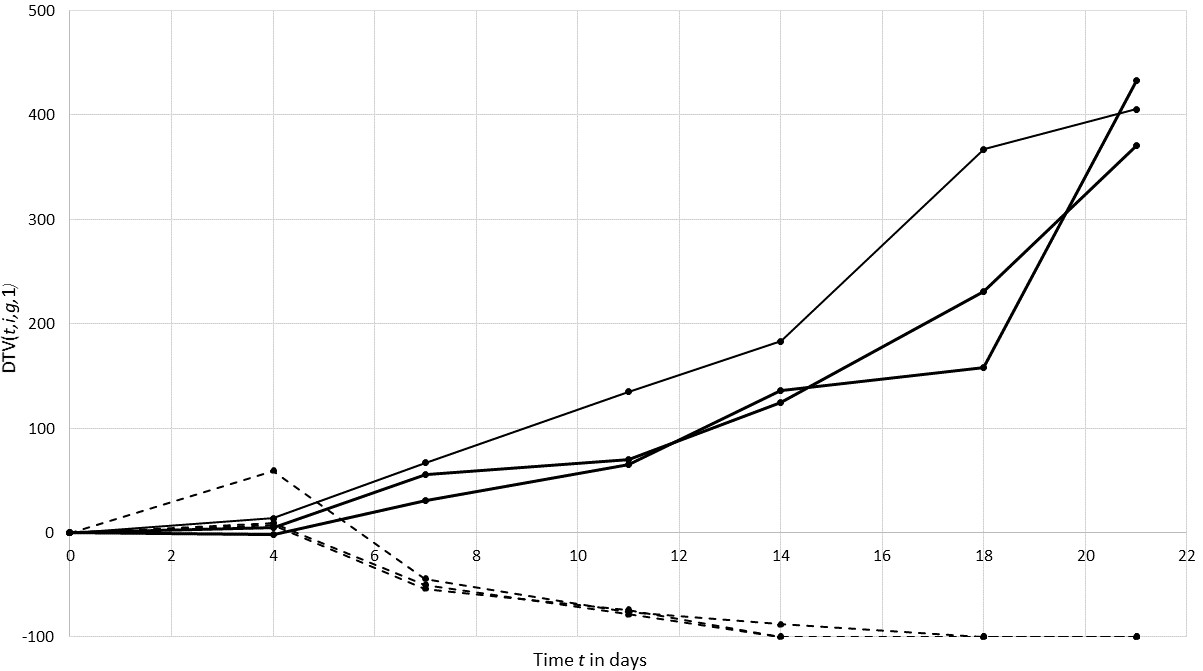


%ΔVol(t)

**Figure S2**. A scatter plot of the Table S2 values for the MDACC lab (*l* =2).


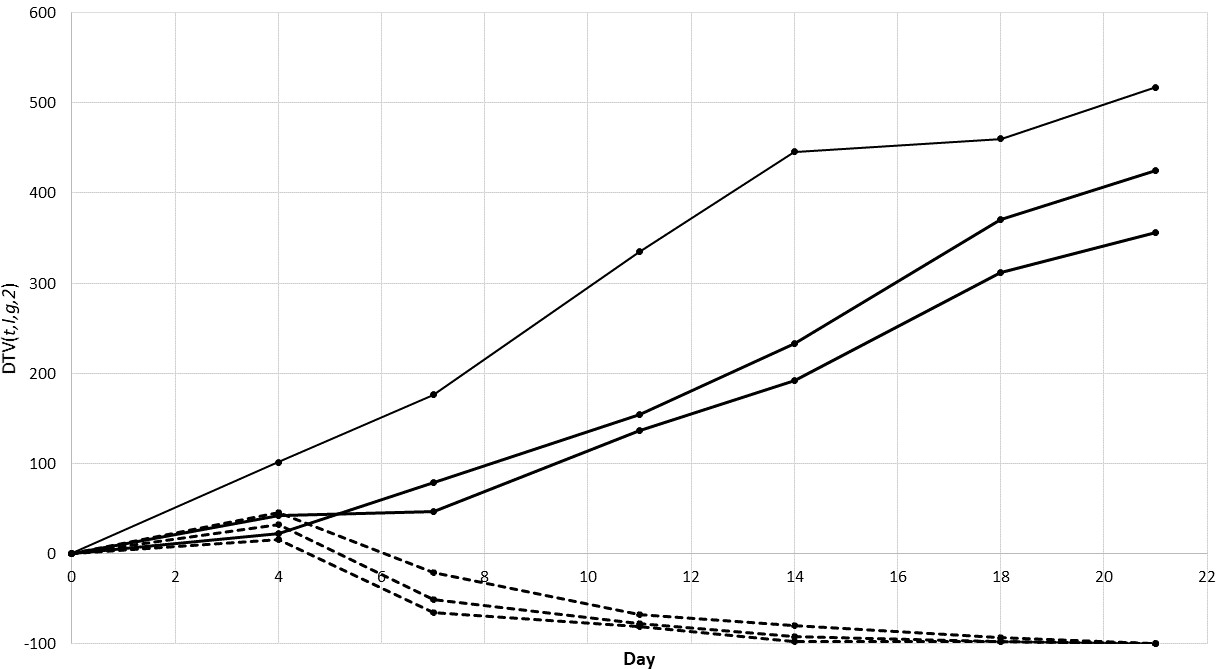


%ΔVol(t)

*Compute p-values for each measure and each lab*

We commented in the Methods of the main section of the paper that, for each measure, there is a corresponding statistic. For all measures and corresponding statistics, we repeat here some of the information presented in the Methods section of the main work. We start with AUCmax and AUC_*t*. These measures have the benefit that they use all the data for a mouse’s empirical tumor growth trajectory.

**AUCmax Measure**: For a given mouse, this measure is the area under the empirical tumor growth trajectory. Because we are dealing with a finite set of points, the integral is simply the sum of the areas of trapezoids. The corresponding statistic is the Welch’s *t*-test, applied to the sample means for the control and treated mice for a given lab. For a given mouse, the lower and upper limits of the integral are *t* = 0 days and *t*_TotalDays_ days, where t_TotalDays_ is the largest day for which %ΔVol(t) exists. Also, we may use the notation max{t(*i,g,l*)} to indicate that we are considering the maximum time (in days) over all times for the *i*^th^ mouse in group *g* of lab *l.* This notation is similar to the notation we use in Tables S1 and S2. As examples, in Table S1 (fictious data), for *i* = 1, *g* = 0, *l* = 2, *t*_TotalDays_ = max{t(*i=*1*,g=*0*,l=*2)} = 15 days. In Table S2 (actual data), for *i* = 3, *g* = 1, *l* = 2, *t*_TotalDays_ = max{t(*i=*3*,g=*1*,l=*2)} = 21 days.

In fact, for Table S2, *t*_TotalDays_ = 21 days for all mice in the control and treated groups, and for both labs. Regarding the Welch’s *t*-test, the null hypothesis is that the difference between the sample means for each group is 0. We use Welch’s test for several of the measures, and we note that there are numerous programs to compute the Welch test. These include the R software package. [4] and Microsoft Excel [4A].

Finally, the formula for computing the integral (i.e., area under the curve) of a mouse’s empirical tumor growth trajectory, the integral from *t*=0 days to *t*_TotalDays_ days is the integral of the set of trapezoids formed by the tumor growth trajectory data points, normalized by *t*_TotalDays_ (this fact noted above). For the *i*^th^ mouse in group *g* of lab *l*, we compute:

$$\int_{t_{1}}^{t_{TotalDays}} empirical curve=\frac{\left[ \sum_{k=1}^{TotalDays} \frac{\%\Delta\mathrm{Vol}(t_{k})+\%\Delta\mathrm{Vol}(t_{k+1})}{2}\times\left( t_{k+1}-t_{k} \right) \right]}{t_{TotalDays}}. (S1)$$

In Formula (S1), the value $t_{k}$ correspond to the day when measurements were recorded, with *N* being the total number of such days. In Table S2, Figures S1 and S2, $t_{1}=0$, $t_{2}=4$, $t_{3}=7$,…,$t_{6}=18$, $t_{7}=t_{TotalDays}=21$. The integral for each mouse is normalized by the largest day, $t_{TotalDays}$. That value is 21 for all mice in Table S2. The normalization in (S1) is only relevant when the $t_{TotalDays}$ values are different for different mice.

Using the values in Table S2, we perform two example calculations: the AUCmax values for the first control mouse in the HCI-BCM lab, and for the first treated mouse in the MDACC lab. Starting with Control Mouse01 (*g* = 0) in Table S2, we compute the AUCmax value using Formula (S1):

$$\begin{matrix} \int_{t_{1}=0}^{t_{TotalDays}=21} empirical curve \\ \\ \begin{matrix} =\left[ \frac{\left( 0+(-1.472) \right)}{2}\times\left( 4-0 \right)+\frac{\left( \left( -1.472 \right)+30.52 \right)}{2}\times\left( 7-4 \right) \right. \\ +\frac{\left( 30.52+65.653 \right)}{2}\times\left( 11-7 \right)+\frac{\left( 65.653+136.408 \right)}{2}\times\left( 14-11 \right) \\ \begin{matrix} +\frac{\left( 136.408+157.9 \right)}{2}\times\left( 18-14 \right) \\ \frac{\left. +\frac{\left( 157.9+432.581 \right)}{2}\times\left( 21-18 \right) \right]}{21}. \end{matrix} \end{matrix} \end{matrix}$$

The sum in brackets is 2010.403. Dividing this quantity by 21 = $t_{TotalDays}$, we conclude that the standardized integral for Control Mouse01 in the HCI-BCM lab, is 95.733. Note that $t_{1}=0$ days.

For the first treated mouse in the MDACC lab Table S2, the AUCmax value using Formula (S1) is:

$$\begin{matrix} \int_{t_{1}=0}^{t_{TotalDays}=21} empirical curve \\ \\ \begin{matrix} =\left[ \frac{\left( 0+45.11 \right)}{2}\times\left( 4-0 \right)+\frac{\left( 45.11+(-20.857) \right)}{2}\times\left( 7-4 \right) \right. \\ +\frac{\left( (-20.857)+(-68.084) \right)}{2}\times\left( 11-7 \right)+\frac{\left( (-68.084)+(-79.4) \right)}{2}\times\left( 14-11 \right) \\ \begin{matrix} +\frac{\left( (-79.4)+(-93.044) \right)}{2}\times\left( 18-14 \right) \\ \frac{\left. +\frac{\left( (-93.044)+(-100) \right)}{2}\times\left( 21-18 \right) \right]}{21}. \end{matrix} \end{matrix} \end{matrix}$$

The sum in brackets is -906.963 (Note that AUCmax values can be negative). Dividing this quantity by 21, we compute that the standardized integral for Treated Mouse01 in the MDACC lab is -43.187.

In Table S3, we report the full set of integrals (Formula S1).

**Table S3**. AUCmax values for all control- and treated-mice in the HCI-BCM and MDACC labs.

| **HCI-BCM (*l* = 1)** | | |
| --- | --- | --- |
| **Control Mouse (*g* = 0)** | **AUCmax** |  |
| *i*  = 1 | 95.733 |  |
| *i*  = 2 | 107.437 |  |
| *i*  = 3 | 156.606 |  |
|  | | |
| **Treated Mouse (*g* = 1)** | **AUCmax** |  |
| *i*  = 1 | -60.253 |  |
| *i*  = 2 | -60.631 |  |
| *i*  = 3 | -48.601 |  |
| **MDACC (*l* = 2)** | | |
| **Control Mouse (*g* = 0)** | **AUCmax** |  |
| *i*  = 1 | 302.283 |  |
| *i*  = 2 | 165.18 |  |
| *i*  = 3 | 147.110 |  |
|  | | |
| **Treated Mouse (*g* = 1)** | **AUCmax** |  |
| *i*  = 1 | -43.187 |  |
| *i*  = 2 | -54.872 |  |
| *i*  = 3 | -61.345 |  |

**Table S3**. All AUCmax values are computed using the Trapezoid Rule (formula S1) for $t_{TotalDays}=21$. The data for calculations come from the empirical tumor growth trajectories in Table S2.

Applying Welch’s test to the values in Table S3, for the two labs, we compute p-values of 0.009 and 0.033 for the HCI-BCM and MDACC labs, respectively.

**AUC_*t* = AUC_*t^’^* (notation for computation purposes) Measure**: The data for this statistic is very similar to that from the AUCmax Measure, with the modification that the denominator in Formula (S1), namely $t_{TotalDays}$, is replaced by a fixed time $t^{'}$, for example $t^{'}$= 18 days. The formula for a given mouse becomes

$$\int_{t_{1}}^{t_{k^{'}}} empirical curve=\frac{\left[ \sum_{k=1}^{k^{'}} \frac{\%\Delta\mathrm{Vol}(t_{k})+\%\Delta\mathrm{Vol}(t_{k+1})}{2}\times\left( t_{k+1}-t_{k} \right) \right]}{t^{'}}. (S2)$$

In equation (S2), the summand $k^{'}$ satisfies $t_{k^{'}}=t^{'}$. The values $t_{k}$ are defined as in the AUCmax Measure section. When ${t^{'}=t}_{TotalDays} is the same$ for all control (*g* = 0) and treated (*g* = 1) mice at a given lab, then the integrals AUC_$t$ and AUCmax are identical. As an example, if we review Table S2, and set $t^{'}=18$, then $k^{'}=6.$ For each mouse, $k^{'}$ may differ. This condition is alright as long as each mouse is measured on day $t^{'}$.

If the largest day for a given mouse is less than $t^{'}$, this mouse is excluded from the statistical calculations. This situation does not apply to the AUCmax measure. The statistic for the AUC_*t* measure, and the corresponding p-value, are computed by evaluating the areas under the empirical tumor growth trajectory for all mice, and then by applying the Welch’s *t*-test to the sample means for the control and treated mice (as with the AUCmax measure). We commented about that the key difference between the AUCmax and AUC_*t* measures is that, for the AUC_*t* measure, each integral is multiplied by the scalar $\frac{1}{t^{'}}$. This scalar may be removed when applying the Welch *t*-test. Thus, the results of the AUC_21 measure statistic are identical to those in Table S3, with the p-values for each lab being provided in the sentences afterwards.

**DTV_*t* Measure**: For a given mouse, this measure is just %∆Vol(t) for a fixed time *t* (days). We use the notation DTV_*t* to indicate that there is a corresponding test statistic. The test statistic is (again), Welch’s t-test applied to the mean values of %∆Vol(t) for controls and treated mice. The null hypothesis is that these means are equal.

Here, we provide an example calculation of the Welch’s test text in an Excel spreadsheet. Consider the %∆Vol(t=21) values for the control mice and treated mice, respectively, in the HCI-BCM lab (*l* = 1) (HCI-BCM). The values come from Table S2. We place these six values into cells A1, A2, and A3 (control) and cells A4, A5, and A6 (treated), respectively.

To compute the Welch’s Test p-value, we use the Excel Function TTEST(array1, array2, tails, type), where array1 is A1:A3, array2 is A4:A6, the number of tails is 2, and the type is 3 (allows for unequal variances among the two groups). We provide a screen shot of the entries in Excel below.

**Figure S3**. Excel cell entries for computing Welch Test p-value with test statistic corresponding to DTV_21 measure for HCI-BCM lab.


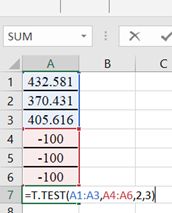


When we apply the TTEST function in this manner, we obtain a p-value of 0.001. Applying the same series of steps to the MDACC data (*l* = 2, Table S2), the Welch *t*-test p-value is 0.008.

**Tumor Growth Index (TGI_*t*) Measure**: In their Supplement, Evrard et al. state (paraphrased) that, to measure anti-tumor activity of the treatment group compared to the control group, they considered the tumor growth treatment-to-control ratio $\gamma\left( t,l \right),$estimated by one way ANOVA focused on time a fixed time $t$. [For the *i*^th^ mouse, 1 ≤ *i* ≤ *I*(*g*,*l*) in group *g* for lab *l*], let $R\left( t \right)=\frac{V\left( t \right)}{V\left( 0 \right)}$ , be the ratio of tumor size at time $t$ (in days) to the size at baseline $\left( t=0 \mathrm{days} \right)$.” We modify this text to conform with our notation. Applying algebra, it is straightforward to show that:

$$R\left( t \right)=\frac{\%\Delta\mathrm{Vol}\left( t \right)}{100}+1, \left( S3 \right).$$

Let $t$, *g*, and $l$ be fixed values. Evrard et al. propose log-transforming the ratios, and fitting the linear model:

$$\ln\left( R\left( t \right) \right)=\alpha+\beta g+\varepsilon\left( i \right), \left( S4 \right).$$

As mentioned above, $t$, *g*, *i*, and $l$ are known. In this model, we specify that the error term for each mouse is normally distributed with mean 0 and a variance term $\sigma^{2}$. Applying Ordinary Least Squares, we can estimate the values $\alpha,\beta,\sigma$ in Equation (S4). These values are the ones we report in our regression results. The null hypothesis in the regression equation (S4) is that $\beta$ is 0. Said another way, for each mouse $i$ in a group *g* at time $t$, the percent relative change $\%\Delta\mathrm{Vol}\left( t \right)$, modulo a random normally distributed error term $\varepsilon(i)$ for the mouse, is a constant function in the group *g*.

We compute the regression statistic as follows. The data are the same as for the DTV_*t* measure, with modification that the outcome variable is $\ln\left( \frac{\%\Delta\mathrm{Vol}\left( t \right)}{100}+1 \right)$, where the input value for each mouse is its group value, *g*. So, the data for this measure are the set of pairs, $\left\{ \left( g,\ln\left( \frac{\%\Delta\mathrm{Vol}\left( t \right)}{100}+1 \right) \right) \right\}$. The group *g* is the input parameter, and the value $\ln\left( \frac{\%\Delta\mathrm{Vol}\left( t \right)}{100}+1 \right)$ is the outcome variable for each mouse. As with previous measures, we set $t$ = 21 days. We present the data for the TGI regression and for labs *l* = 1 and *l* = 2 in Table S4.

**Table S4**. Values $\%\Delta\mathrm{Vol}\left( t \right)$ for *t* equal to 21 days, transformed for TGI_*t* regression.

| **Lab** | **Mouse** | **Group (*g*)** | $\boldsymbol{\%\Delta}\mathbf{Vol}\left( \boldsymbol{21} \right)$ | $\ln\left( \frac{\boldsymbol{\%\Delta}\mathbf{Vol}\left( \boldsymbol{21} \right)}{\boldsymbol{100}}\boldsymbol{+1} \right)$ |
| --- | --- | --- | --- | --- |
| *l* = 1 (HCI-BCM) | *i*  = 1 | 0 | 432.581 | 1.673 |
|  | *i*  = 2 | 0 | 370.431 | 1.548 |
|  | *i*  = 3 | 0 | 405.616 | 1.621 |
|  |  | | | |
|  | *i*  = 1 | 1 | -99 | -4.605 |
|  | *i*  = 2 | 1 | -99 | -4.605 |
|  | *i*  = 3 | 1 | -99 | -4.605 |
|  | | | | |
| *l* = 2 (MDACC) | *i*  = 1 | 0 | 517.302 | 1.820 |
|  | *i*  = 2 | 0 | 425.392 | 1.659 |
|  | *i*  = 3 | 0 | 356.102 | 1.518 |
|  |  |  |  |  |
|  | *i*  = 1 | 1 | -99 | -4.605 |
|  | *i*  = 2 | 1 | -99 | -4.605 |
|  | *i*  = 3 | 1 | -99 | -4.605 |

**Table S4**. Here, the values -99 for $\%\Delta\mathrm{Vol}\left( 21 \right)$ are used, rather than the values -100 that are present in Table S2. The reason is that, if $\%\Delta\mathrm{Vol}\left( 21 \right)$ = -100, then $\ln\left( \frac{\%\Delta\mathrm{Vol}\left( 21 \right)}{100}+1 \right)$= ln(0), or negative infinity.

We perform the regression using the Regression option under “Data Analysis” in the Excel Data tab. We present the results in Tables S5A and 5B below.

**Table S5A.** Regression analysis for TGI Measure with data at time *t* = 21 days, *l* = 1 (HCI-BCM).

| **Regression Statistics** | |
| --- | --- |
| Multiple R | 1 |
| R Square | 1 |
| Adjusted R Square | 1 |
| StdError | 0.044 |
| Observations | 6 |

| **ANOVA** | | | | | |
| --- | --- | --- | --- | --- | --- |
|  | **DF** | **SumSq** | **MeanSq** | **F Stat** | **P-value** |
| Regression | 1 | 58.015 | 58.015 | 29879.814 | 6.719E-09 |
| Residual | 4 | 0.008 | 0.002 |  | |
| Total | 5 | 58.023 |  |  |  |

| **Variable** | **Coefficients** | **StdError** | **t Stat** | **P-value** | **Lower 95%** | **Upper 95%** |
| --- | --- | --- | --- | --- | --- | --- |
| Intercept (*α*) | 1.614 | 0.025 | 63.438 | 3.698E-07 | 1.543 | 1.685 |
| Group (*g*) | -6.219 | 0.036 | -172.858 | 6.719E-09 | -6.319 | -6.119 |

**Table S5B.** Regression analysis for TGI Measure with data at time *t* = 21 days, *l* = 2 (MDACC).

| **Regression Statistics** | |
| --- | --- |
| Multiple R | 1 |
| R Square | 0.999 |
| Adjusted R Square | 0.999 |
| Standard Error | 0.107 |
| Observations | 6 |

| **ANOVA** | | | | | |
| --- | --- | --- | --- | --- | --- |
|  | DF | SumSq | MeanSq | F Stat | P-value |
| Regression | 1 | 58.983 | 58.983 | 5144.491 | 2.264E-07 |
| Residual | 4 | 0.046 | 0.011 |  | |
| Total | 5 | 59.029 |  |  |  |

| **Variable** | **Coefficients** | **StdError** | **t Stat** | **P-value** | **Lower 95%** | **Upper 95%** |
| --- | --- | --- | --- | --- | --- | --- |
| Intercept (*α*) | 1.666 | 0.062 | 26.942 | 1.128E-05 | 1.494 | 1.837 |
| Group (*g*) | -6.271 | 0.087 | -71.725 | 2.264E-07 | -6.513 | -6.028 |

**Legend for Tables S5A and S5B**. In these boxed tables, the p-value for the regression is the same as the p-value for the *t*-test of the Group indicator. The abbreviations SumSq, MeanSq, and StdError stand for Sum of Squares, Mean Square for Error, and StdError respectively. Also, the headings “Lower 95%” and “Upper 95%” stand for the lower and upper 95% confidence intervals for the variables.

The main results of the regression analysis are that the p-values corresponding to the null hypothesis that the coefficient for each Group variable (*g*) in each table is 0, are substantially lower than 0.05. They are even lower than the most stringent significance level we consider in this work, namely 0.001. The coefficient estimates are -6.219 and -6.271 for the HCI-BCM lab (*l* = 1; Table S5A) and the MDACC lab (*l* = 2; Table S5B), respectively. These results are consistent with the comments by Evrard et al. that, under the alternative hypothesis, the Group coefficients should be less than 0.

Given the TGI measure results, we conclude that the drug treatment is inhibitory for tumor growth at the two labs.

**Progression-free Survival (**$\boldsymbol{PFS}_{\boldsymbol{\delta}}$**) Measure**: This measure involves survival analysis methodology. For the *i*^th^ mouse in group *g* and lab *l*, there are three key pieces of information t:

1. The definition of an Event;
2. The largest time *t* (measured in days),$t\_max$, for which the *i*^th^ mouse’s $\%\Delta\mathrm{Vol}\left( t \right)$ value exists (The term $t\_max$ is defined in the AUCmax Measure section);
3. For mouse *i*, a determination of the Time To Event. This information is drawn from the mouse’s empirical tumor group trajectory. We determine this value below.
4. The parameter $\delta$; We paraphrase from Evrard et al.’s text [5].
   1. Progression-free survival [for our mouse *i*] is defined as the shortest time $t^{*}$ when the $\%\Delta\mathrm{Vol}\left( t \right)$ meets or exceeds 𝛿 percent. That is,

$t^{*}\left( i \right)=\min\left\{ t \right|\%\Delta\mathrm{Vol}\left( t \right)\geq\delta\times100, 0\leq t\leq t_{TotalDays}\}$. (S5)

For example, 𝛿 equal to 0.5 corresponds to the shortest time *t* such that $\%\Delta\mathrm{Vol}\left( t \right)$ is greater than 0.5 × 100 = 50, 𝛿 equal to 2 the shortest time for which $\%\Delta\mathrm{Vol}\left( t \right)$ ≥ 200, and 𝛿 equal to 4 the shortest time for which $\%\Delta\mathrm{Vol}\left( t \right)$ ≥ 400.

If $t^{*}\left( i \right)\leq t_{TotalDays}$, then the Time to Event is $t^{*}(i)$. If there is no day *t* that satisfies Equation (S5), or if the mouse drops out of the study before $t_{TotalDays}$ for some reason other than it achieving the time to event $t^{*}\left( i \right)$, then we say that the mouse is censored.

The general framework for this analysis is the Kaplan Meier survival analysis. The test statistic is the log rank test, and the null hypothesis is that the two survival distributions are the same.

**Table S6**. Time To Event Values and Censor classification for all mice in Control and Treated Groups for both labs when multiplier $\delta$ is 50%.

| **Lab** | **Mouse** | **Group (*g*)** | **Time to Event** $\boldsymbol{t}^{\boldsymbol{*}}$ **(in Days)** | **Censored?** |
| --- | --- | --- | --- | --- |
| *l* = 1 (HCI-BCM) | *i*  = 1 | 0 | 11 | No |
|  | *i*  = 2 | 0 | 7 | No |
|  | *i*  = 3 | 0 | 7 | No |
|  |  | | | |
|  | *i*  = 1 | 1 | 21 | Yes |
|  | *i*  = 2 | 1 | 21 | Yes |
|  | *i*  = 3 | 1 | 4 | No |
|  | | | | |
| *l* = 2 (MDACC) | *i*  = 1 | 0 | 4 | No |
|  | *i*  = 2 | 0 | 7 | No |
|  | *i*  = 3 | 0 | 11 | No |
|  |  |  |  |  |
|  | *i*  = 1 | 1 | 21 | Yes |
|  | *i*  = 2 | 1 | 21 | Yes |
|  | *i*  = 3 | 1 | 21 | Yes |

**Figure S3A**. Kaplan-Meier Survival Curves for HCI-BCM data.


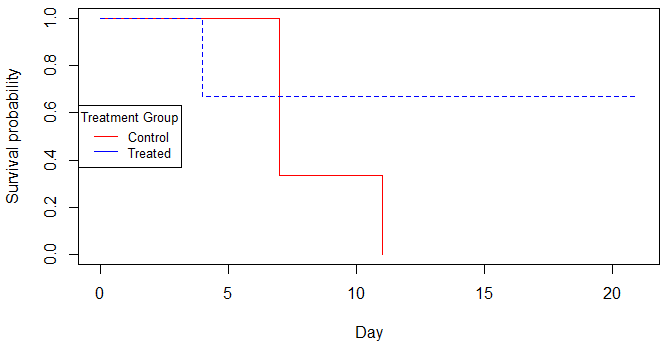


**Figure S3A.** The data for this figure come from Table S2. The threshold value 𝛿 for this analysis is 0.5. That is, we declare that an event has occurred at Day $t^{*}$ for mouse $i$ in group $g$ when $\%\Delta\mathrm{Vol}\left( t \right)\geq50$, and $t^{*}$ is the earliest day for which this inequality is satisfied. These values are provided in Table S6. The plot is generated using the “survival” package in the R software program.

The Chi-Square Goodness of Fit Test for Survival Data tests the null hypothesis that the survival distributions are the same among the different groups (two in this case). For the HCI-BCM data, we obtain a Chi-Square value of 1.123, with a corresponding p-value of 0.289. Under the null hypothesis, the test statistic has a central chi-square distribution with *g* – 1 degrees of freedom, where g = the number of groups considered in the analysis. Here, g = 2; Control and Treated, so the degrees of freedom is 1.

We perform the same analysis for the MDACC data (Table S2). The survival plot and conclusions are below.

**Figure S3B**. Kaplan-Meier Survival Curves for MDACC data.


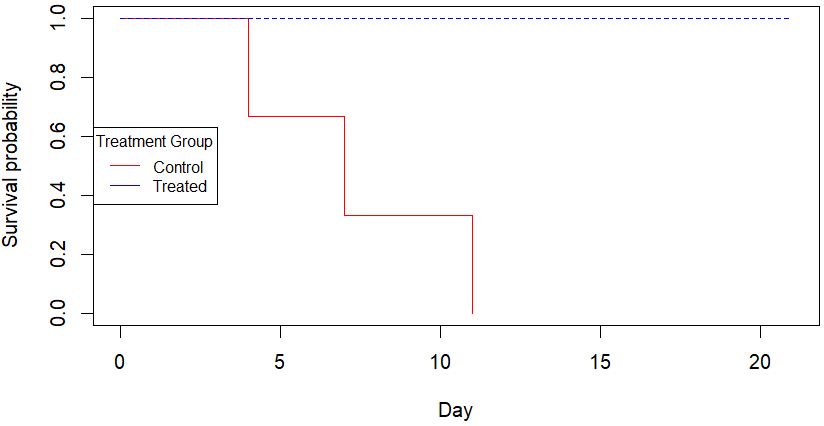


**Figure S3B.** This plot is created using the information from Table S2B. The threshold value 𝛿 for this analysis is 0.5. That is, we declare that an event has occurred at Day $t^{*}$ for mouse $i$ in group $g$ when $\%\Delta\mathrm{Vol}\left( t \right)\geq50$, and $t^{*}$ is the earliest day for which this inequality is satisfied. These values are provided in Table S6. The plot is generated using the “survival” package in the R software program.

For the MDACC data, we obtain a Chi-Square value of 5.052, with a corresponding p-value of 0.025. As with the HCI-BCM data, the degrees of freedom for the Chi-Square Goodness of Fit Test is 1.

*Creating the p-value and q-value table (Basis Table)*

Having applied the appropriate statistical analyses for all measures for both labs, we can summarize the results in a table of p-values.

**Table S7.** Measure-specific p-values for two research labs.

| **Measure** | **HCI-BMC**  **P-value** | **MDACC**  **P-value** |
| --- | --- | --- |
| AUCmax | 0.009 | 0.033 |
| AUC_21 | 0.009 | 0.033 |
| DTV_21 | 0.001 | 0.008 |
| TGI_21 | 6.719E-09 | 2.264E-07 |
| PFS_0.5_ | 0.289 | 0.025 |

**Table S7.** For each of the measures, we provide the corresponding test statistic. The p-values are determined applying that statistic to the appropriate data (also described for each measure). Fisher’s method is a meta-analysis technique for combining multiple p-values into a single p-value. We shall discuss this method further below. As mentioned before, HCI-BCM is the first lab (*l* = 1) and MDACC is the second lab (*l* = 2).

Table S7 is precisely what we need to create the basis table.

*Necessary statistical methods*

Several of our screening tests, defined below, make use of a meta-analysis and/or multiple statistical tests applied to the same data set. There are two statistical methods we use to address these issues.

*Meta-analysis with Fisher’s Method*

When there are several independent p-values for a given statistical test, as there are for any of the measures and the multiple research labs, a meta-analysis helps us to make a more informed decision about the drug treatment. As compared to individual p-values, that may provide conflicting results as to the drug treatment inhibition status, the results of a meta-analysis provide a single p-value reflecting the nature of the treatment over all labs.

For this work, we make use of Fisher’s Method, a meta-analysis method that allows us to combine p-values from independent experiments into a single p-value. In this data set, to perform our meta-analysis for a specific measure, we compute a function of the two p-values corresponding to that measure (Table S7). Let the two p-values be labeled $p_{\_m,1}$, $p_{\_m,2}$, for an arbitrary measure *m*, $1\leq m\leq M$. In general, the Fisher’s Method statistic (function) is:

$$X^{2}=\sum_{l=1}^{L} -2\ln\left( p_{\_m,l} \right),$$

and the null hypothesis for Fisher’s Method is that all of the separate null hypotheses (for the different labs) are true. The statistic $X^{2}$ follows a central chi-square distribution with 2*L* degrees of freedom under the null hypothesis.

The alternative hypothesis is that, for at least one of the labs, the null hypothesis is not true; that is, for that lab, results indicate that the drug treatment is inhibitory for the tumor models. In this supplement, *L* = 2. In the main manuscript, *L* = 5.

*Correction for multiple testing*

For some of the screening tests, we combine the p-values from multiple measures to make a decision regarding the drug treatment inhibition status. Given the fact there are multiple p-values considered, we need to make a correction for multiple testing. Failure to do so may lead to inflation in the false positive rate. In other words, if the null hypothesis is true, then at the $\alpha$ significance level, the probability that we reject the null hypothesis is greater than $\alpha$ when there is no multiple-test correction. Practically speaking, inflation in the false positive rate means that we conclude that a treatment is inhibitory when it is not.

Here we apply Storey’s q-value method (described in main text) to correct for multiple testing.

*Determining those p-values that are significant after multiple-test correction Q-value method*

The algorithm is as follows.

1. Let $\left\{ p_{\_1,l},p_{\_2,l},\ldots,p_{\_M,l} \right\}$ be the set of p-values in the *l*^th^ column of a table like Table S7.
2. Sort the list (#1) and re-write as $\left\{ p_{(M,l)},p_{(M-1,l)},\ldots,p_{(1,l)} \right\}$ where $p_{(M,l)}\geq p_{\left( M-1,l \right)}\ldots\geq p_{(1,l)}$.
3. Consider the function:
   1. $q_{(j,l)}=\left\{ \begin{matrix} p_{(M,l)},j=M \\ \min\left( q_{(j+1,l)},\frac{M}{j}p_{(j,l)} \right),1\leq j<M \end{matrix}. \right.$
4. For those q-values $q_{(j,l)}$satisfying$q_{(j,l)}\leq\alpha$, for a specified significance level, we say that the unsorted p-value $p_{\_j',l}=q_{(j,l)}$ is significant at the $\alpha$ level after correction for multiple testing.

With this information we can create the following.

**Table S8**. Basis table using information from Table S7.

|  | **Measure** | **HCI-BCM**  **P-value** | **MDACC**  **P-value** | **Fisher’s Method** |
| --- | --- | --- | --- | --- |
| p-values | AUCmax | 0.009 | 0.033 | 0.003 |
|  | AUC_21 | 0.009 | 0.033 | 0.003 |
|  | DTV_21 | 0.001 | 0.008 | 1.00E-04 |
|  | TGI_21 | 6.72E-09 | 2.26E-07 | 5.34E-14 |
|  | PFS_0.5_ | 0.289 | 0.025 | 0.043 |
| q-values | AUCmax | 0.011 | 0.033 | 0.004 |
|  | AUC_21 | 0.011 | 0.033 | 0.004 |
|  | DTV_21 | 0.0025 | 0.02 | 2.5E-04 |
|  | TGI_21 | 3.36E-08 | 1.13E-06 | 2.67E-13 |
|  | PFS_0.5_ | 0.289 | 0.033 | 0.043 |

*Screening tests*

We define a screening test as function whose input information is the Basis Table of p-values (e.g., S8) and a significance level, and whose outcome is a drug treatment classification. There are two possible classifications: the drug treatment is inhibitory, or not inhibitory. In what follows, we describe four screening tests. For each test, we describe how we determine the inhibition-classification of the drug treatment.

Because we have ground truth classification for each tumor type, we can determine the sensitivity or specificity for each of the screening tests. Because we are working with data from the Completely Responsive tumor type (Table S2), in this section, can calculate sensitivity.

1. Single-Measure-Single-Lab Test (SMSL)

Given Table S8, we observe that there are 5 measures $\times$ 2 labs = 10 p-values. We exclude the Fisher’s Method column for now. With the “Single Measure for a Single lab” (SMSL) screening test, a drug treatment is evaluated for one lab and for one measure. Drug-treatment inhibition classification is determined by the (single measure, single lab) p-value (upper left, clear cells in Table S8). If the p-value is less than the specified significance level, we classify the drug treatment as inhibitory. For example, if the significance level *α* is 0.05, then the proportion of p-values that are less than *α* is 9/10, or 90%, in Table S8. The only p-value greater than 0.05 is for the (PFS_0.5_, HCI-BCM) with a p-value of 0.289.

If we consider a more stringent significance level of *α* = 0.01, then the proportion of times the drug is classified as inhibitory drops to 60%. The AUCmax and AUC_21 p-values for the MDACC lab no longer indicate that the drug is inhibitory, and the two PFS_0.5_ measure p-values show the same result. That is, for four p-values, our decision is that the drug treatment is not inhibitory.

1. Single-Measure-Numerous-Lab Test (SMNL)

The “Single Measure for Numerous labs” (SMNL) screening test is an extension of the SMSL test. Rather than consider the p-value for a single measure and a single lab, we combine the p-values across all labs for a given measure through the Fisher’s Method. Again, a benefit of Fisher’s Method is that it can reduce or remove conflicting results as to the drug treatment inhibition status from SMSL tests, and provide a more accurate classification. The null hypothesis is that each of the measure-p-values for each lab is drawn from a U[0,1] distribution.

For this screening test, drug-treatment inhibition classification is determined by the (single measure, all-labs) Fisher’s Method p-value. If the p-value is less than the specified significance level, we classify the drug treatment as inhibitory. In Table S8, we see that there are 5 Fisher’s Method p-values (upper right, light gray cells), corresponding to the five measures. Like the SMSL test, our decision rule for the SMNL screening test is that the drug treatment is inhibitory if the Fisher’s Method p-value for a given measure is less than *α*. That is, a decision is based on a pair (single measure, single p-value), where the single p-value in this case is the Fisher’s Method p-value.

Let us consider the Basis Table S8 again. We set *α* to 0.05. It follows the proportion of Fisher’s Method p-values that are less than *α* is 5/5, or 100%. That is, the SMNL test result is that the drug treatment is inhibitory for every measure at the *α* = 0.05 level. This proportion is 10% greater than the SMSL proportion for the same significance level.

For *α* equal to 0.01, the proportion of Fisher’s Method p-values that are less than *α* is 4/5, or 80%. The Fisher’s Method p-value for the PFS_0.5_ measure is 0.043, greater than the significance level and therefore we classify the drug treatment as non-inhibitory.

1. Numerous-Measure-Single-Lab Test (NMSL)

A third screening test is the “Numerous Measure for a Single lab” Test (NMSL). For a single lab in Table S8, we gather the five p-values corresponding to the five measures for that lab. We compute the q-values corresponding to the same lab’s p-values. We compute the proportion of q-values (lower left, dark gray cells) that are less than *α*, a specified significance level. If at least one measure-q-value satisfies the inequality, we declare that the drug treatment is inhibitory for the lab. We have confirmed through extensive simulations that specifying at least one measure-q-value as being significant after correction maintains the correct false positive rate.

For the significance levels 0.10, 0.05, 0.01, and 0.001, there is always at least one q-value for each lab in Table S8 that is less than the respective significance level (TGI_21). Therefore, we specify that the drug treatment is inhibitory for each lab, a proportion of 2/2 = 1.0. Hence, the sensitivity is 1.0 for all significance levels.

1. Numerous-Measure-Numerous-Lab Test (NMNL)

The last screening test is the “Numerous Measure for Numerous labs” test (NMNL). This test is a combination of the SMNL test that considers the set of single-measure Fisher’s Method p-values (Table S8; the first column are the measures, the last column are the p-values) and the NMSL test, that considers p-values for all measures at a single lab. In the NMNL test, the term “lab” is replaced by the Fisher’s Method p-values (e.g., Table S8, last column). For these p-values, we apply the Q-value multiple-test correction. For the NMNL screening test, drug-treatment inhibition classification is determined by the (all measures, all-labs) Fisher’s Method p-values. If at least one Fisher’s Method q-value (lower right, steel blue cells) is less than a specified significance level, we declare that the drug treatment classification is inhibitory. As with the NMSL screening test, we performed large numbers of simulations to verify that this classification rule maintains the correct type I error rate.

Because the TGI_21 q-value for the Fisher’s Method p-values is 2.67E-13 (Table S8), we specify that the drug treatment is inhibitory for all significance levels.

Summarizing results for the different screening tests, we determine the following sensitivities for the four screening tests at the significance levels 0.10, 0.05, 0.01, and 0.001.

**Table S9**. Sensitivities for the four screening tests given the basis table S8.

| **Significance level** | **0.1** | **0.05** | **0.01** | **0.001** |
| --- | --- | --- | --- | --- |
| SMSL | 0.9 | 0.9 | 0.6 | 0.3 |
| SMNL | 1 | 1 | 0.8 | 0.4 |
| NMSL | 1 | 1 | 1 | 1 |
| NMNL | 1 | 1 | 1 | 1 |

We discussed some examples above. We can consider other examples here. At a significance level of 0.001, the sensitivity of the SMSL test is 0.3. That is, only three of the ten p-values in Table S8 are less than or equal to 0.001. So, the sensitivity is 3/10 = 0.30. For the SMNL test, at a significance level of 0.10, all Fisher p-values (right most, top five entries of Table S8) are less than 0.10. Therefore, the sensitivity is 5/5 = 1.0.

In general, for the HCI-BCM and MDACC data sets, the NMSL and NMNL screening tests are optimal. Sensitivity is 1.0 for all significance levels.

*Computing Bootstrap Confidence Intervals*

The sensitivities in Table S9 are those for a single set of data (the actual data). We do not know the theoretical distributions for the screening tests, so we cannot employ the standard methods for determining mean, median, and 95% significance levels. Such values indicate what screening tests may be optimal. As an alternative, we apply Bootstrap resampling using the actual data sets. To generate bootstrap resamples, we apply a stratified resampling with replacement. Specifically, for a given group (control, treated), and a specific lab, we randomly create a bootstrap sample by randomly drawing one of the three mice, and replacing the actual mouse’s data by the bootstrap mouse’s data. We present examples below in Tables S10 through S13 (four bootstrap samples). The lab column indicates the lab for which the mice’s measures were computed, and the group indicates the treatment status (Group = 0 for control, Group = 1 for treated) for a given mouse. Note that terms like “Mouse 01” represent different mice if the group and/or lab value is different. A more complete description of the data and notation may be found in Table S2.

As examples of bootstrap resampling, in Table S10, HCI-BCM lab Control Mouse 02 (group = 0) in the original data set is replaced by Control Mouse 03 (third row). In the same table, HCI-BCM lab Treated Mouse 03 is replaced by Treated Mouse 02.

As another example, in Table S11, MDACC lab treated Mouse 01 (group = 1) is *not* replaced by another mouse. That is, in the bootstrap sample, the original mouse has not changed (11^th^ row of that table).

An important point is that a given mouse within a specific lab and group has an associated set of measure values (each table, Columns 5 – 10). One can check that these values are provided in Table S2.

**Table S10** Bootstrap 1

| **Trial** | **Lab** | **Group** | **Mouse** | **DTV21** | **AUC21** | **AUCmax** | **logRit** | **PFS_T** | **PFS_E** |
| --- | --- | --- | --- | --- | --- | --- | --- | --- | --- |
| 1 | HCI-BCM | 0 | Mouse01 | 432.58 | 2010.40 | 95.73 | 1.67 | 21 | 1 |
| 1 | HCI-BCM | 0 | Mouse03 | 405.62 | 3288.73 | 156.61 | 1.62 | 21 | 1 |
| 1 | HCI-BCM | 0 | Mouse01 | 432.58 | 2010.40 | 95.73 | 1.67 | 21 | 1 |
| 1 | HCI-BCM | 1 | Mouse03 | -100.00 | -1020.62 | -48.60 | -4.61 | 21 | 0 |
| 1 | HCI-BCM | 1 | Mouse03 | -100.00 | -1020.62 | -48.60 | -4.61 | 21 | 0 |
| 1 | HCI-BCM | 1 | Mouse02 | -100.00 | -1273.25 | -60.63 | -4.61 | 21 | 0 |
| 1 | MDACC | 0 | Mouse03 | 356.10 | 3089.32 | 147.11 | 1.52 | 21 | 0 |
| 1 | MDACC | 0 | Mouse02 | 425.39 | 3642.94 | 173.47 | 1.66 | 21 | 1 |
| 1 | MDACC | 0 | Mouse02 | 425.39 | 3642.94 | 173.47 | 1.66 | 21 | 1 |
| 1 | MDACC | 1 | Mouse03 | -100.00 | -1288.25 | -61.35 | -4.61 | 21 | 0 |
| 1 | MDACC | 1 | Mouse02 | -100.00 | -1152.32 | -54.87 | -4.61 | 21 | 0 |
| 1 | MDACC | 1 | Mouse03 | -100.00 | -1288.25 | -61.35 | -4.61 | 21 | 0 |

**Table S11** Bootstrap 2.

| **Trial** | **Lab** | **Group** | **Mouse** | **DTV21** | **AUC21** | **AUCmax** | **logRit** | **PFS_T** | **PFS_E** |
| --- | --- | --- | --- | --- | --- | --- | --- | --- | --- |
| 2 | HCI-BCM | 0 | Mouse02 | 370.43 | 2256.19 | 107.44 | 1.55 | 21 | 0 |
| 2 | HCI-BCM | 0 | Mouse03 | 405.62 | 3288.73 | 156.61 | 1.62 | 21 | 1 |
| 2 | HCI-BCM | 0 | Mouse03 | 405.62 | 3288.73 | 156.61 | 1.62 | 21 | 1 |
| 2 | HCI-BCM | 1 | Mouse02 | -100.00 | -1273.25 | -60.63 | -4.61 | 21 | 0 |
| 2 | HCI-BCM | 1 | Mouse02 | -100.00 | -1273.25 | -60.63 | -4.61 | 21 | 0 |
| 2 | HCI-BCM | 1 | Mouse01 | -100.00 | -1265.32 | -60.25 | -4.61 | 21 | 0 |
| 2 | MDACC | 0 | Mouse03 | 356.10 | 3089.32 | 147.11 | 1.52 | 21 | 0 |
| 2 | MDACC | 0 | Mouse02 | 425.39 | 3642.94 | 173.47 | 1.66 | 21 | 1 |
| 2 | MDACC | 0 | Mouse02 | 425.39 | 3642.94 | 173.47 | 1.66 | 21 | 1 |
| 2 | MDACC | 1 | Mouse01 | -100.00 | -906.93 | -43.19 | -4.61 | 21 | 0 |
| 2 | MDACC | 1 | Mouse01 | -100.00 | -906.93 | -43.19 | -4.61 | 21 | 0 |
| 2 | MDACC | 1 | Mouse02 | -100.00 | -1152.32 | -54.87 | -4.61 | 21 | 0 |

**Table S12** Bootstrap 3.

| **Trial** | **Lab** | **Group** | **Mouse** | **DTV21** | **AUC21** | **AUCmax** | **logRit** | **PFS_T** | **PFS_E** |
| --- | --- | --- | --- | --- | --- | --- | --- | --- | --- |
| 3 | HCI-BCM | 0 | Mouse01 | 432.58 | 2010.40 | 95.73 | 1.67 | 21 | 1 |
| 3 | HCI-BCM | 0 | Mouse03 | 405.62 | 3288.73 | 156.61 | 1.62 | 21 | 1 |
| 3 | HCI-BCM | 0 | Mouse03 | 405.62 | 3288.73 | 156.61 | 1.62 | 21 | 1 |
| 3 | HCI-BCM | 1 | Mouse02 | -100.00 | -1273.25 | -60.63 | -4.61 | 21 | 0 |
| 3 | HCI-BCM | 1 | Mouse03 | -100.00 | -1020.62 | -48.60 | -4.61 | 21 | 0 |
| 3 | HCI-BCM | 1 | Mouse01 | -100.00 | -1265.32 | -60.25 | -4.61 | 21 | 0 |
| 3 | MDACC | 0 | Mouse02 | 425.39 | 3642.94 | 173.47 | 1.66 | 21 | 1 |
| 3 | MDACC | 0 | Mouse03 | 356.10 | 3089.32 | 147.11 | 1.52 | 21 | 0 |
| 3 | MDACC | 0 | Mouse01 | 517.30 | 6089.29 | 289.97 | 1.82 | 14 | 1 |
| 3 | MDACC | 1 | Mouse01 | -100.00 | -906.93 | -43.19 | -4.61 | 21 | 0 |
| 3 | MDACC | 1 | Mouse02 | -100.00 | -1152.32 | -54.87 | -4.61 | 21 | 0 |
| 3 | MDACC | 1 | Mouse02 | -100.00 | -1152.32 | -54.87 | -4.61 | 21 | 0 |

**Table S13** Bootstrap 4.

| **Trial** | **Lab** | **Group** | **Mouse** | **DTV21** | **AUC21** | **AUCmax** | **logRit** | **PFS_T** | **PFS_E** |
| --- | --- | --- | --- | --- | --- | --- | --- | --- | --- |
| 4 | HCI-BCM | 0 | Mouse01 | 432.58 | 2010.40 | 95.73 | 1.67 | 21 | 1 |
| 4 | HCI-BCM | 0 | Mouse02 | 370.43 | 2256.19 | 107.44 | 1.55 | 21 | 0 |
| 4 | HCI-BCM | 0 | Mouse03 | 405.62 | 3288.73 | 156.61 | 1.62 | 21 | 1 |
| 4 | HCI-BCM | 1 | Mouse01 | -100.00 | -1265.32 | -60.25 | -4.61 | 21 | 0 |
| 4 | HCI-BCM | 1 | Mouse03 | -100.00 | -1020.62 | -48.60 | -4.61 | 21 | 0 |
| 4 | HCI-BCM | 1 | Mouse02 | -100.00 | -1273.25 | -60.63 | -4.61 | 21 | 0 |
| 4 | MDACC | 0 | Mouse02 | 425.39 | 3642.94 | 173.47 | 1.66 | 21 | 1 |
| 4 | MDACC | 0 | Mouse01 | 517.30 | 6089.29 | 289.97 | 1.82 | 14 | 1 |
| 4 | MDACC | 0 | Mouse01 | 517.30 | 6089.29 | 289.97 | 1.82 | 14 | 1 |
| 4 | MDACC | 1 | Mouse03 | -100.00 | -1288.25 | -61.35 | -4.61 | 21 | 0 |
| 4 | MDACC | 1 | Mouse01 | -100.00 | -906.93 | -43.19 | -4.61 | 21 | 0 |
| 4 | MDACC | 1 | Mouse02 | -100.00 | -1152.32 | -54.87 | -4.61 | 21 | 0 |

From these tables, we can calculate thr tables of SMSL p-values for each trial. We can also compute the Fisher p-values and q-values (data not shown). P-values greater than 0.05 are highlighted in bold.

**Table S14** Bootstrap resampling basis table.

| **Trial** | **Lab** | **DTV21** | **AUC21** | **AUCmax** | **TGI21** | **PFS4** |
| --- | --- | --- | --- | --- | --- | --- |
| 1 | HCI-BCM | 2.95E-04 | 1.17E-02 | 1.17E-02 | 1.76E-10 | 2.53E-02 |
| 1 | MDACC | 2.11E-03 | 8.89E-04 | 8.89E-04 | 9.91E-09 | **1.14E-01** |
|  |  |  |  |  |  |  |
| 2 | HCI-BCM | 5.63E-04 | 6.60E-03 | 6.60E-03 | 6.78E-10 | **1.14E-01** |
| 2 | MDACC | 2.11E-03 | 3.51E-04 | 3.51E-04 | 9.91E-09 | **1.14E-01** |
|  |  |  |  |  |  |  |
| 3 | HCI-BCM | 3.05E-04 | 8.90E-03 | 8.90E-03 | 1.78E-10 | 2.53E-02 |
| 3 | MDACC | 7.59E-03 | 2.77E-02 | 2.77E-02 | 1.13E-07 | **1.16E-01** |
|  |  |  |  |  |  |  |
| 4 | HCI-BCM | 1.28E-03 | 8.67E-03 | 8.67E-03 | 3.36E-09 | **1.14E-01** |
| 4 | MDACC | 2.72E-03 | 1.46E-02 | 1.46E-02 | 1.52E-08 | 2.24E-02 |

We can check that the sensitivities for the drug screening tests at the 0.05 significance level are those values provided in Table

**Table S15** Screening test sensitivity across bootstrap resampling trials for α = 0.05.

| **Trial** | **SMSL** | **SMNL** | **NMSL** | **NMNL** |
| --- | --- | --- | --- | --- |
| 1 | 0.9 | 1.0 | 1.0 | 1.0 |
| 2 | 0.8 | 0.8 | 1.0 | 1.0 |
| 3 | 0.9 | 1.0 | 1.0 | 1.0 |
| 4 | 0.9 | 1.0 | 1.0 | 1.0 |

Four bootstrap resamplings are not sufficient to calculate a 95% confidence interval, so for the sake of illustration we repeat each result 2500 times to determine a set of 10,000 screening test results. Figure S4 below shows a histogram of the number of bootstrap samples out of 10,000 that return a given sensitivity score for each screening test.

**Figure S4**. Histogram of number of bootstrap samples with a given sensitivity for each screening test.

We sort the sensitivity values in order from smallest to largest. The 250^th^ value, representing the 2.5^th^ percentile, becomes the lower end of the 95% CI. The 9750^th^ value, representing the 97.5^th^ percentile, becomes the upper end of the 95% CI. These can be visualized as a clustered bar chart, as in Figure S5 below.

**Figure S5**. Median sensitivities with 95% CIs for bootstrap data.

Like our findings in Table S9 for the actual data, we may conclude that, for this data set, the optimal screening tests are the NMSL and NMNL tests.

References

1. Igl BW, Konig IR, Ziegler A: **What do we mean by 'replication' and 'validation' in genome-wide association studies?** *Hum Hered* 2009, **67**(1):66-68.

2. Evrard YA, Srivastava A, Randjelovic J, Doroshow JH, Dean DA, 2nd, Morris JS, Chuang JH, Consortium NCIP: **Systematic Establishment of Robustness and Standards in Patient-Derived Xenograft Experiments and Analysis**. *Cancer Res* 2020, **80**(11):2286-2297.

3. Gordon D, Axelrod DE: **A reliable method to determine which candidate chemotherapeutic drugs effectively inhibit tumor growth in patient-derived xenografts (PDX) in single mouse trials**. *Cancer Chemother Pharmacol* 2019, **84**(6):1167-1178.

4. R Development Core Team (2012): **R: A language and environment for statistical computing**. *R Foundation for Statistical Computing*, **Vienna, Austria. ISBN 3-900051-07-0, URL** [**http://www.R-project.org/**](http://www.R-project.org/).

4A. Microsoft Corporation. (2018). Microsoft Excel. Accessed from https://office.microsoft.com/excel

5. Evrard YA, Srivastava A, Randjelovic J, Doroshow JH, Dean DA, Morris JS, Chuang JH: **Systematic Establishment of Robustness and Standards in Patient-Derived Xenograft Experiments and Analysis**. *Cancer Research* 2020, **80**(11):2286-2297.

6. Benjamini Y, Hochberg Y: **Controlling the False Discovery Rate: a practical and powerful approach to multiple testing**. *J Roy Stat Soc B* 1995, **57**(1):289-300.
