# Supplementary material for: Improved drug-screening tests of candidate anti-cancer drugs in patient-derived xenografts through use of numerous measures of tumor growth determined in multiple independent laboratories
Source: PLoS One. 2025 Jun 18;20(6):e0324141. doi: 10.1371/journal.pone.0324141 (PMC12176186; doi:10.1371/journal.pone.0324141)
Supplement: Attachment — Submitted filename: Response-to-Reviewers.docx [file pone.0324141.s008.docx]

April 11, 2025

To The Editorial Board of PLOS One:

Thank you for the providing us with the thoughtful comments from two reviewers. We are grateful for the opportunity to submit a revised version of our manuscript, incorporating the changes indicated by the reviewers. We include the reviewers’ comments, followed our responses in italics. The responses indicate the changes we have made in the manuscript text, tables and figures provided in this resubmission.

*We start by reporting that we have modified our Abstract so that it less than PLOS One’s Word Count limit of 300 words.*

**Reviewer 1:**

The manuscript is scientifically sound considering the need to develop newer drug screening tests in patient-derived xenografts. It is well drafted and the statistical analysis have been performed effectively.

*Authors’ response: We very much appreciate the reviewer’s kind words.*

However, there are certain modifications to be made to increase the quality and readership of the article.

1. The authors should include the abbreviations below all the tables. For example, in Tables 7, 9 and 10, the abbreviations of the corresponding labs are suggested to be incorporated in the revised manuscript.

*Authors’ response: As per the reviewer’s recommendation, we have added abbreviations to all tables.*

2. The authors can also mention if there were any similarities in both the datasets obtained from different labs.

*Authors’ response: We appreciate the reviewer’s recommendation. We have added the following paragraph to the Methods section, after the sub-heading “Threshold selection on BRCA-paclitaxel data” and right before the sub-heading “Statistical measures used”:*

*“Similarities among the Evrard et al. and Gao et al. data sets are that they both considered PDX trials, both had candidate drug treatments, the data generated from the tumor models and drug treatments consisted of longitudinal tumor growth data, specifically the measure of tumor size in each mouse at given time points. Because of this experimental design, we were able to apply the same statistical tests to data from each set, and subsequently, compute the outcomes for each drug screening test.”*

3. It would be ideal to summarize the sensitivity and specificities of the screening methods of meta-analysis in a single table under Results or Conclusion section.

*Authors’ response: We thank the reviewer for this suggestion. We have added a new Table 10 summarizing Sensitivity and Specificity for the SMNL and NMNL drug screening tests. Also, we have added the following text:*

*“A result that comes from studying Table 10 is that, for sensitivity, SMNL and NMNL screening tests are superior. For every significance level and both datasets, the median sensitivity is 1.0, and the 95% confidence interval is (1.0,1.0), meaning 95% of the bootstrap samples have a sensitivity of 1.0. For specificity, optimal results occurred for the 0.01 and 0.001 significance levels for the NMNL (both datasets) and for 0.001 for the SMNL (apart from the 0.001 significance level, Gao et al. dataset). It follows that, to obtain the highest sensitivity and specificity jointly, we select the NMNL screening test and specify a significance level of 0.01..”*

4. The figures look somewhat ‘blurry’ and quality of the figures can be enhanced.

*Authors’ response: We applied the following protocol to produce high quality figures.*

1. *We opened the original Powerpoint figure.*
2. *We went to the File->Options->Advanced page.*
3. *We selected “Do not compress” and “Default …:High Quality”.*
4. *We saved the figure in the .tiff format.*

**Reviewer 2**:

The present work is entitled: “Improved drug-screening tests of candidate anti-cancer drugs in patient-derived xenografts through the use of numerous measures of tumor growth determined in multiple independent laboratories.” The purpose of this work is to develop methods for evaluating the results of preclinical drug screening tests. Several different measures of drug inhibition of tumor growth in patient-derived xenografts (PDX) are compared, and the benefit of determining inhibition in different laboratories are compared. The work is interesting and suitable for publication in the PLOS One after making minor revisions. I suggest the authors to make the necessary corrections as suggested below.

1. Rewrite the keywords in alphabetical order. Additionally, add the other keywords preclinical, in vivo and in vitro.

*Authors’ response: We thank the reviewer for this recommendation. We have added the suggested keywords and have alphabetized the overall list.*

2. Check the manuscript for grammatical and typographical errors. For example, keep the word et al, in vivo, in vitro in italics, and while writing the word figure “F” must be capital letter.

*Authors’ response: We have performed a thorough search, correcting typographical errors, italicizing all occurrences of in vitro, in vivo, and et al., and replacing all lowercase f’s in the word “figure” with capital F.*

3. Table legends of all the tables mentioned in the manuscript have to be kept above the table.

*Authors’ response: We have made the changes indicated by the reviewer. Also, figure titles are now in bold, and titles and legends are below each figure.*

4. Double check the entire documents, different typos are present.

*Authors’ response: We addressed this point in Item #2.*

5. To improve the quality of manuscript, add some of the recent findings related to anti-cancer in introduction and results.

*We thank the reviewer for this excellent suggestion. In response, we have added a paragraph to the Introduction section, reviewing some of the most relevant articles suggested by Reviewer 2. The paragraph starts with “Testing newly developed drugs for tumor inhibition...” We have added appropriate citations to the list of references.*

Again, we thank you very much for your consideration of our work. We sincerely hope that you will find this revised work worthy of publication.

Please do not hesitate to contact me with any comments or questions you may have regarding this work. My phone, fax, and e-mail are listed above.

Sincerely,

Derek Gordon, Ph.D.

Associate Professor

(signing for all co-authors)
